# Making the case for the governance of (digital public) health futures

**DOI:** 10.1093/eurpub/ckac129.152

**Published:** 2022-10-25

**Authors:** B Wong

**Affiliations:** 1 GHFutures2030 Commission Secretariat, Geneva, Switzerland; 2 EUPHA-DH

## Abstract

Digital and data tools are fundamentally changing approaches to health and the design of health systems, but governance models have neither followed nor kept up with the pace of innovation. In response to this challenge, The Lancet & Financial Times Commission on Governing health futures 2030: Growing up in a digital world explores the convergence of digital health, artificial intelligence, and other frontier technologies with universal health coverage to support attaining the SDG 3. Children and young people are crucial groups requiring particular attention to ensure that no one is left behind in achieving universal health coverage and SDG 3 amidst the digital transformation in health. Today, there are 1.8 billion people between the ages of 10 and 24 - the largest youth population in history - 90 percent of whom live in developing countries. This cohort represents an unprecedented powerhouse of human potential and digital engagement that could transform health to reach sustainable development goals. This presentation introduces several key findings from the Commission's report which pertain to the governance of (digital public) health futures amidst digital transformations in health. It will highlight how human-centred approaches to health are vital to navigating the digital transformations and maximising their benefits for population health and well-being. Further, it will provide an action plan for meaningful youth engagement in the design, development, implementation, and evaluation of digital public health policies, programmes, and services.

